# Negativity-bias in forming beliefs about own abilities

**DOI:** 10.1038/s41598-019-50821-w

**Published:** 2019-10-08

**Authors:** Laura Müller-Pinzler, Nora Czekalla, Annalina V. Mayer, David S. Stolz, Valeria Gazzola, Christian Keysers, Frieder M. Paulus, Sören Krach

**Affiliations:** 1 Department of Psychiatry and Psychotherapy, Social Neuroscience Lab, University of Lübeck, Ratzeburger Allee 160, D-23538 Lübeck, Germany; 2 Department of Psychiatry and Psychotherapy, Translational Psychiatry Unit (TPU), University of Lübeck, Ratzeburger Allee 160, D-23538 Lübeck, Germany; 3 Social Brain Lab, Netherlands Institute for Neuroscience, KNAW, Meibergdreef 47, NL-1105BA Amsterdam, The Netherlands; 4 Department of Psychology, University of Amsterdam, Nieuwe Achtergracht 116, NL-1018 WV Amsterdam, The Netherlands

**Keywords:** Learning algorithms, Human behaviour

## Abstract

During everyday interactions people constantly receive feedback on their behavior, which shapes their beliefs about themselves. While classic studies in the field of social learning suggest that people have a tendency to learn better from good news (positivity bias) when they perceive little opportunities to immediately improve their own performance, we show updating is biased towards negative information when participants perceive the opportunity to adapt their performance during learning. In three consecutive experiments we applied a computational modeling approach on the subjects’ learning behavior and reveal the negativity bias was specific for learning about own compared to others’ performances and was modulated by prior beliefs about the self, i.e. stronger negativity bias in individuals lower in self-esteem. Social anxiety affected self-related negativity biases only when individuals were exposed to a judging audience thereby potentially explaining the persistence of negative self-images in socially anxious individuals which commonly surfaces in social settings. Self-related belief formation is therefore surprisingly negatively biased in situations suggesting opportunities to improve and this bias is shaped by trait differences in self-esteem and social anxiety.

## Introduction

People examine their own thoughts, behavior and their efficacy, making “corrective adjustments if necessary”^1^. They develop beliefs about their abilities^2^, i.e. the innate and formed capacities that enable them to perform particular tasks successfully, which become strong motivators for subsequent behaviors and are thus fundamental for well-being^3–6^. Already during the formative periods of development, children’s beliefs in their academic efficacy, e.g. mathematical or language self-concepts, have the most pervasive direct impact on their judgment of their later occupational efficacy^7^. Not only in childhood, but throughout the entire lifespan self-related beliefs thus shape future performance and behavior^1,8,9^. Though intensive research on the influence of peers and societal norms on self-efficacy beliefs has been conducted^10,11^, surprisingly little is known about the learning mechanisms underlying the formation of self-related ability beliefs^3^.

With the present studies we aim to find answers for three central questions: First, how do people process feedback on their abilities and form beliefs about themselves? Second, how do differences in personality impact this process and finally, how does the social context shape such learning?

Previous studies demonstrate that we update our beliefs in response to the feedback we receive. Rather than integrating feedback in a way that results in an accurate representation of the world studies show that self-related information is not perceived objectively^12,13^. The perception of self-related feedback is influenced by various motivational factors. Positive beliefs have an intrinsic value^13^ as individuals strive to be viewed in a positive and self-serving light^14^. This culminates in a robust and often replicated positivity bias for learning of self-related information^13,15–18^. Particularly, people show increased updates of their self-related beliefs when information was better than expected (positive prediction error) compared to when information was worse than expected (negative prediction error)^15^. However, all of these studies have focused on self-related belief updating by confronting people with feedback concerning aspects of the self that are often perceived as rather difficult to change (e.g. IQ, likelihood of dying from a disease)^15–18^. While people might be able to improve in those aspects by long-term training or preventive health strategies they cannot be directly modified by the agent in the course of the experiment. Does this positivity bias thus also apply to the many cases in which the recipient of feedback can immediately alter the behavior that has been appraised? Humans often have the opportunity to improve^3,19^. For example, when processing information about their job or school performance (“Am I good at my job?”; “Am I a good student?”) or sociability (“Am I a likeable person?”), they can directly act to improve them (e.g. by putting more effort in the next task at work or school or acting more prosocially during the next social interaction). There might thus be a difference in how people update their own ability beliefs based on the presence or absence of the perceived opportunity to improve. Situations suggesting little opportunity for improvement may encourage a positivity bias to regulate mood, whilst those suggesting significant opportunities to improve abilities may encourage the processing of negative information to focus effort where it is most needed^4,7,20,21^. In order to fully understand how beliefs are formed and updated it is therefore important to explore whether positivity biases also apply in situations suggesting opportunities for change, for instance by providing performance feedback while people develop a novel skill as compared to facing rather unchangeable facts.

An important related question is how people differ in how they form beliefs about their abilities. The functional value of stable self-efficacy beliefs in contrast to “the self-handicapping costs of nagging self-doubts about one’s capabilities” has often been discussed^1^. For example, studies in the field of developmental and educational psychology continuously demonstrate that already at very young age a child’s fundamental lack of belief in his/her own ability to achieve – while not lacking in actual abilities – consistently tempers their ambition^7^. Self-related beliefs have the potential to imbue perception and interpretation of feedback (e.g. confirming prior beliefs)^22,23^ and thereby impact consecutive behavior (i.e. task persistence and effort)^8,9,24^. The impact of negative self-related beliefs might be even more detrimental in individuals with mental health conditions like depression^25^ and social anxiety disorder^26–28^. In such clinical conditions negative beliefs can lead to reduced intrinsic motivation or avoidance behavior and thus exacerbate a self-perpetuating cycle of negative self-related thoughts^29–31^. It is therefore important to consider interindividual differences in personality to unravel potential maladaptive learning biases and mechanisms specific for self-related beliefs.

The social context itself plays another crucial role with respect to the formation of self-related beliefs. Being in public changes how people perceive and evaluate their own behavior^32,33^ and it is argued that the presence of other individuals increases arousal, implicating behavioral consequences^34,35^. Humans do not only differ in their general self-related beliefs but also in their specific beliefs to be capable of coping with public situations. Especially socially anxious individuals fear social evaluation and feel unable to make the desired impression in a social context^36–38^. Thus, the social context elicits negative cognitions and emotions that are thought to shape self-efficacy beliefs^1^. Our aim is thus to examine how prior beliefs about the self impact how individuals learn about their own abilities in a performance situation and how the social context in which individuals perform and receive feedback, e.g. feedback provided under observation or in privacy, shapes self-related learning.

**Figure 1:**
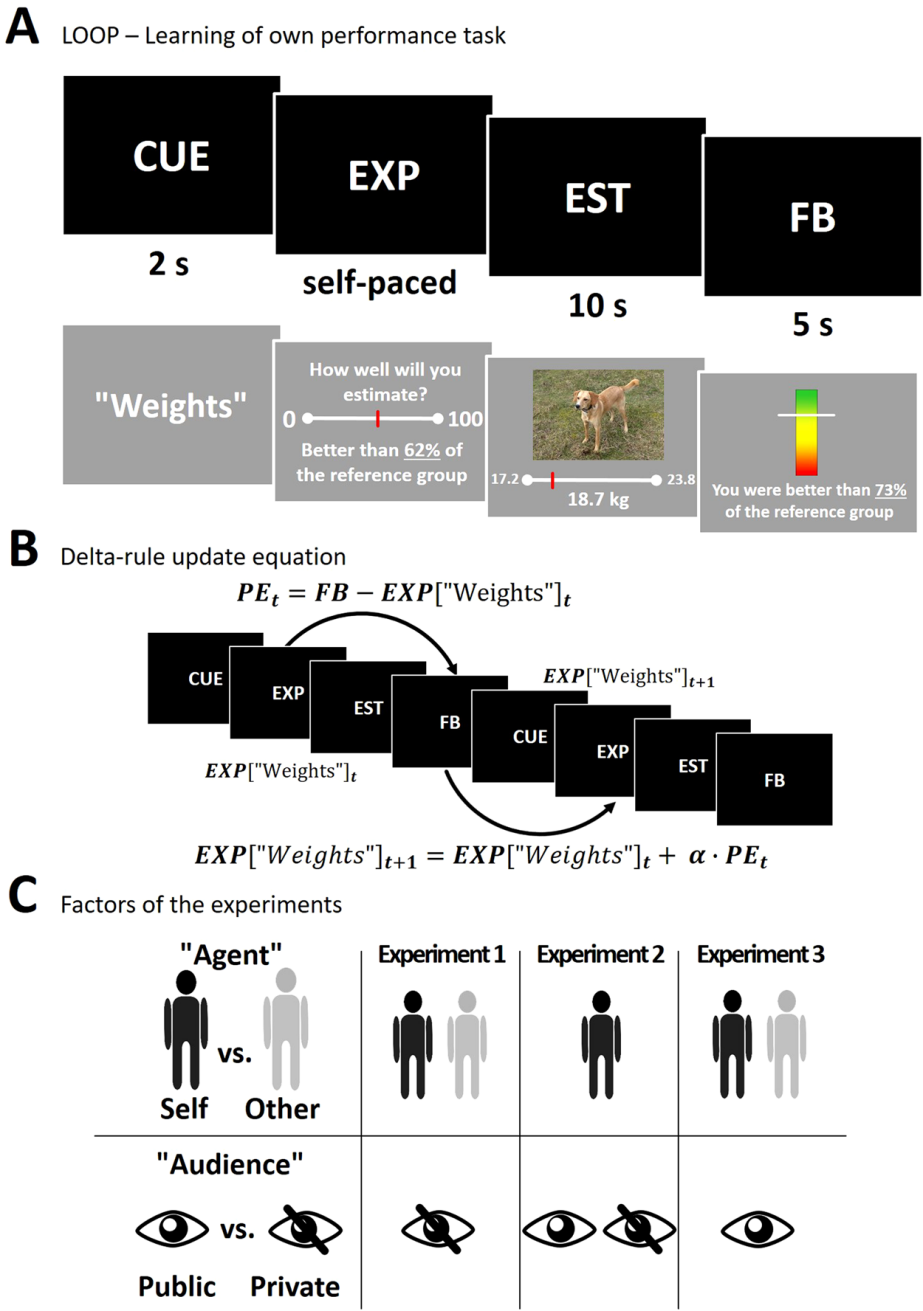
Trial sequence, modeling of learning behavior, and experimental factors of the experiments. (**A)** A cue (CUE) in the beginning of each trial indicated the following estimation category. After providing their performance expectation ratings (EXP) participants received an estimation question (EST), followed by the corresponding performance feedback (FB). (**B)** EXP ratings were modeled by means of Rescorla-Wagner delta-rule update equations with different learning rates (α, see Methods) taking into account trial-by-trial prediction errors (PE_t_) in response to the provided FB. (**C)** In three experiments we assessed the impact of two experimental factors. The “Agent” was manipulated within subjects in the Agent-LOOP task in experiments 1 and 3 and the “Audience” was manipulated in a between-subject design in the Audience-LOOP task (experiment 2) as well as between the Private and the Public group of the Agent-LOOP task (experiment 1 vs experiment 3).

Introducing the “Learning of own performance” (LOOP) task (see Fig. 1), we examined in three studies how people update self-related beliefs in an ability domain that is novel for them, i.e. cognitive estimation (such as estimating the weights of animals), unlike e.g. mathematical skills for which people hold strong and rigid prior beliefs about their potential capabilities. We implemented a performance-feedback-loop that mimics everyday life performance situations. Inferring prediction error (PE) learning rates by fitting computational learning models we assessed the modulatory influence of self-relatedness, prior beliefs, and the social context on belief updating. Our hypotheses were that when learning about the self, the weight of self-related negative feedback would be increased, because negative feedback gains specific importance for behavior regulation by signaling a demand to increase task-related effort. This led us to predict that this effect would be absent for non-self-related feedback. Second, we assessed whether prior beliefs about the self modulate self-related belief-formation. Here, our expectation was that self-esteem and social anxiety would shift updating behavior in line with a confirmation bias. As suggested by prior studies this implies that individuals higher in social anxiety would show increased biases towards negative information^39,40^. Third, we expected the negativity bias in social anxiety to be augmented by a social context, i.e. the presence of an evaluative audience, which triggers social fear related cognition and behavior.

## Results

### Experimental design

#### Experiment 1: Agent-LOOP

The LOOP task formed the main frame for three separate experiments. In experiment 1 we implemented the LOOP task manipulating the “Agent” of the estimation performance to assess how participants learned about themselves (Self condition) compared to learning about another (Other condition). In doing so we aimed to provide an answer for our first main question: how do people process feedback on their abilities and form beliefs about themselves (as compared to learning about another person as a control condition)? Participants were invited in pairs to a study on cognitive estimation. The estimation tasks involved answering estimation questions while receiving manipulated relative performance feedback for each question. Participants took turns in performing the task themselves or allegedly observing the other person performing, while continuously indicating the expected performance (EXP ratings) for the upcoming trial in a High Ability condition and a Low Ability condition (resulting in four feedback conditions: Agent condition (Self vs Other) × Ability condition (High Ability vs Low Ability); see Methods for a detailed description of the task)). Our second question, how differences in personality impact self-related learning, was investigated across all three experiments. In experiments 1 we assessed a person’s general sense of self-competence or self-esteem via the Self-Description Questionnaire before participants were involved in the estimation task (SDQ-III^41^). We also assessed social interaction anxiety via the Social Interaction Anxiety Scale (SIAS^42^). For more details on the sample’s questionnaire data see Supplementary Table S1.

#### Experiment 2: Audience-LOOP

In experiment 2 we implemented another version of the LOOP task, to answer our third main question: how does the social context shape self-related learning? We now assessed the impact of the presence of an audience, i.e. being in public or not, on self-related learning in a between-subject design (Fig. 1C). Participants were invited alone and were randomly assigned to one of two experimental groups (Private vs Public group; see Methods section for further details) resulting in four experimental conditions (Ability condition (High Ability vs Low Ability) × Audience group (Private vs Public)). Here again social interaction anxiety scores served to assess how differences in personality impact self-related learning and specifically how this is modulated by the social context.

#### Experiment 3: replication and extension

We conducted a third experiment again implementing the Agent-LOOP task (experiment 1), while introducing publicity in a more minimal fashion compared to the Audience-LOOP (experiment 2). With this task variant we aimed to replicate the previous findings as well as to provide evidence for the specificity of the audience effect for self-related learning compared to learning about another person. Self-esteem and social interaction anxiety scores were assessed as described above.

### Model free behavioral analysis

**Figure 2:**
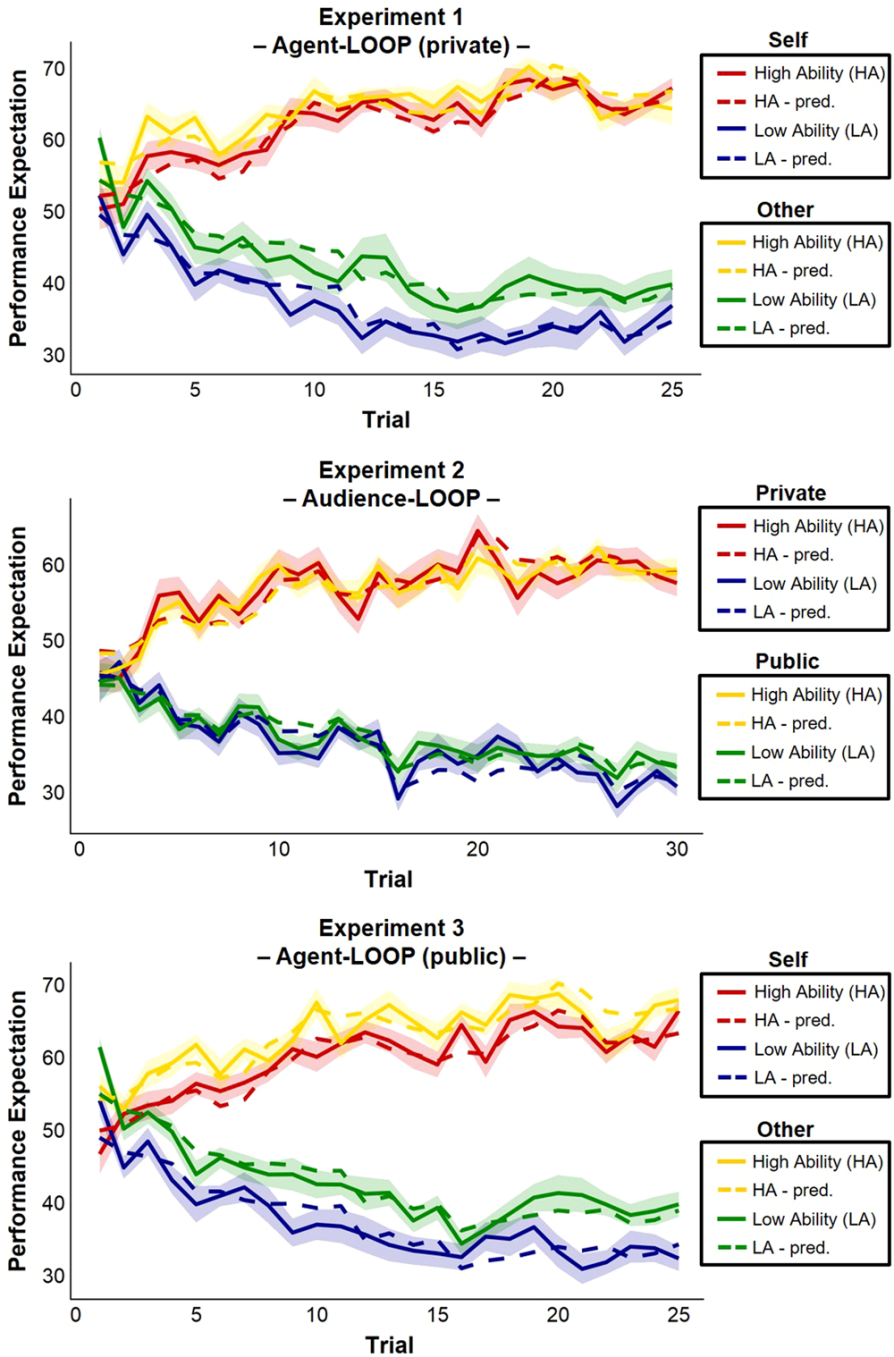
Predicted and actual performance expectation ratings across time. The behavioral data of the three experiments (averaged across subjects) indicate that participants adapted their performance expectation ratings (solid lines) to the provided feedback, thus learning about their allegedly distinct performance levels in the two ability conditions. In the Agent-LOOP (top and bottom) participants evaluated their own performance more negatively than the other’s performance. Our valence specific learning model captured the participants’ behavior for all experiments. Shaded areas represent the standard errors for the actual performance expectations for each trial. Predicted data (pred.) are represented by the dashed lines.

We first performed a model free analysis to capture the basic effects we see in our behavioral data (see Methods section for further details). For the Agent-LOOP in experiment 1 the Trial × Ability condition × Agent condition ANOVA revealed a significant main effect of Ability condition (*F*_*(1*,2*2)*_ = 215.26, *p* < 0.001) and interaction of Trial × Ability condition (*F*_*(24,528)*_ = 31.43, *p* < 0.001) reflecting that participants adapted their EXP ratings over time according to the feedback provided in each Ability condition (see Fig. 2). The significant main effect of Agent condition (*F*_*(1,22)*_ = 15.24, *p* = 0.001) and interaction of Agent condition × Ability condition (*F*_*(1,22)*_ = 4.65, *p* = 0.042) both indicate that participants evaluated their own performance more negatively than the other’s performance, specifically in the Low Ability condition. There was no significant interaction of Trial × Agent condition × Ability condition (*F*_*(24,528)*_ = 0.99, *p* = 0.476).

For the Audience-LOOP (experiment 2) the Trial × Ability condition × Audience ANOVA revealed a significant main effect of Ability condition (*F*_*(1,57)*_ = 261.56, *p* < 0.001) and interaction of Trial × Ability condition (*F*_*(29,1653)*_ = 39.84, *p* < 0.001) indicating that participants adapted their EXP ratings over time, while there was no significant impact of the Audience on EXP ratings (main effect of Audience: *F*_*(1,57)*_ = 0.09, *p* = 0.767; Audience × Ability condition: *F*_*(1,57)*_ = 0.15, *p* = 0.700; Audience × Ability condition × Trial: *F*_*(29,1653)*_ = 1.00, *p* = 0.467).

For the public version of the Agent-LOOP in experiment 3 we replicated the findings of experiment 1 (main effect Ability condition: *F*_*(1,28)*_ = 182.99, *p* < 0.001; interaction of Trial × Ability condition: (*F*_*(24,672)*_ = 36.80, *p* < 0.001). Similarly, there was a significant main effect of Agent condition (*F*_*(1,28)*_ = 18.49, *p* < 0.001), while the interaction of Agent condition × Ability condition (*F*_*(1,28)*_ = 2.18, *p* = 0.151) and the Trial × Agent condition × Ability condition interaction (*F*_*(24,672)*_ = 1.12, *p* = 0.316) failed to reach significance, indicating that participants evaluated their own performance more negatively than the other’s performance independently of the ability condition. The combined analysis of the public and private Agent-LOOP (experiment 1 and 3) confirmed the results of the Audience-LOOP by showing that Audience did not have any significant effects also with regards to the additional Agent condition (all *ps* > 0.439). The remaining effects stayed consistent with the separate analyses of experiment 1 (for more details see Supplementary Results).

### Model selection for computational models of learning behavior

**Figure 3:**
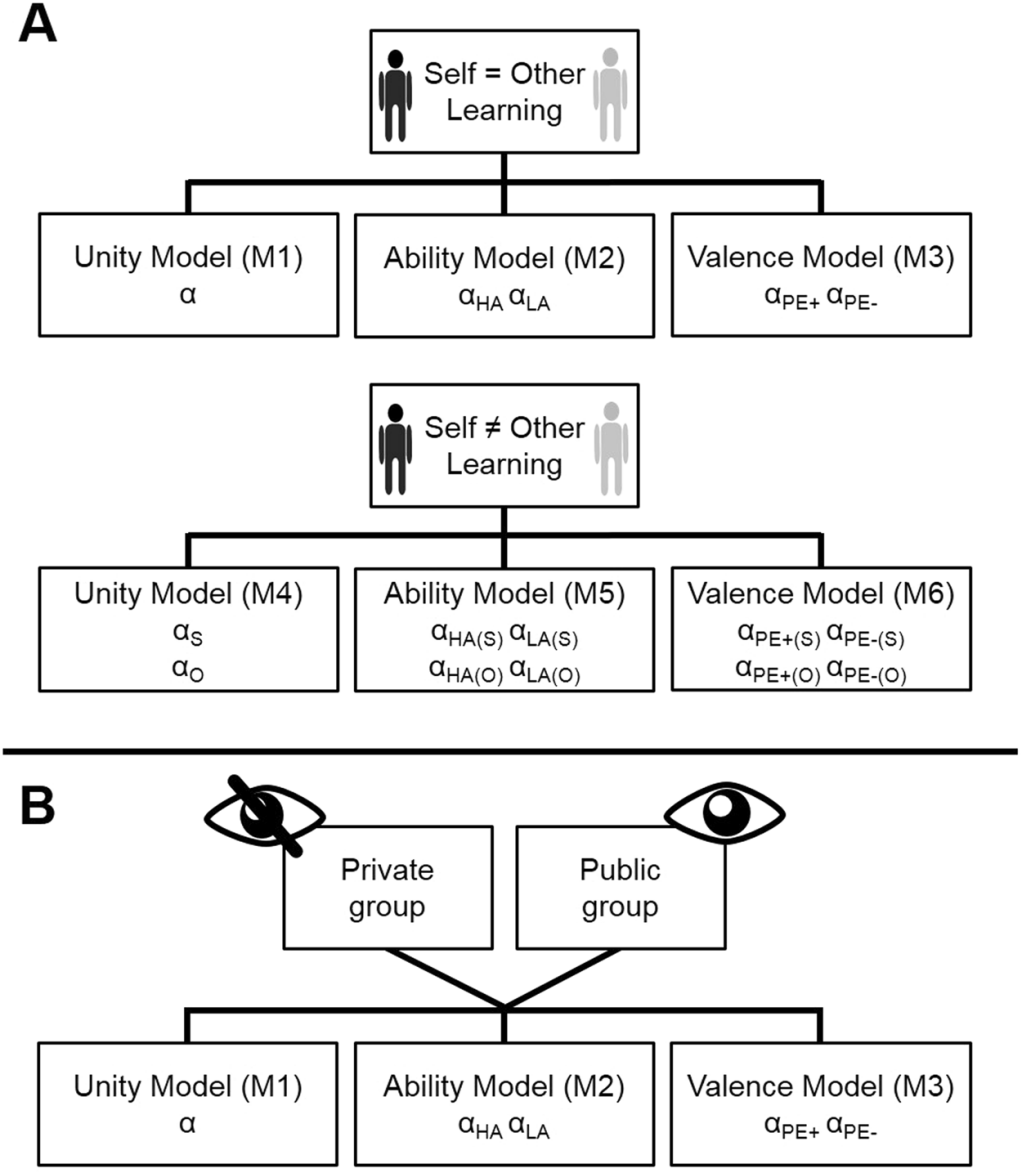
Structure of the model space for the three experiments. (**A)** In the Agent-LOOP task (experiments 1 and 3) we distinguished two factors impacting learning rates: the agent (Self vs Other) and the impact (no impact: Unity Model) of the ability condition (Ability Model) or valence (Valence Model). (**B)** In the Audience-LOOP task the impact of the ability condition or valence on learning rates was assessed within the Private and the Public group separately. For a more detailed description of the model space including initial values for the performance expectations see Supplementary Methods.

To see whether a learning model can capture the participants’ behavior and allows us to summarize the data using principled parameters such as learning rates, we performed a model comparison (see Fig. 3). Our model space contained three main models varying with regards to their assumptions about biased updating behavior when learning about the self (see Fig. 3). The simplest learning model used one single learning rate for the whole behavioral time course for each participant, thus not assuming any learning biases [EXP_t+1_ = EXP_t_ + α_Uni_ PE_t_, while PE_t_ = FB_t_ − EXP_t_; Unity Model]. The second model, the Ability Model, contained a separate learning rate for each of the ability conditions, assuming that participants would show different updating behavior in the High Ability condition (α_HA_) vs Low Ability condition (α_LA_). The third model, the Valence Model, included separate learning rates for positive PEs (α_PE+_) vs negative PEs (α_PE−_) across both ability conditions, thus suggesting that the valence (positive vs negative) of the PE biases self-related learning rather than the ability condition itself. In the Agent-LOOP task (experiments 1 and 3) the distinction between learning about oneself vs another person was introduced as a second factor in the model space resulting in three additional models. Model 4 corresponded to the Unity Model with separate learning rates for the self (α_Uni(S)_) and the other person (α_Uni(O)_). Model 5 was the extension of the Ability Model distinguishing between learning about the self (α_HA(S)_, α_LA(S)_) and the other person (α_HA(O)_, α_LA(O)_), resulting in four different learning rates. Model 6 extended the Valence Model by separate learning rates for oneself (α_PE+(S)_, α_PE−(S)_) and the other person (α_PE+(O)_, α_PE−(O)_). To test if the participants’ EXP ratings could be better explained in terms of prediction error learning as compared to stable assumptions in each Ability condition, we included a simple Mean Model with a mean value for each task condition (two values for the Audience-LOOP (Model 4) and four values for the Agent-LOOP (Model 7)).

For the Agent-LOOP – implementing model comparison across experiment 1 and 3 – the Valence Model with separate learning rates for Self vs Other (Model 6) received the highest sum PSIS-LOO score out of all models (for all PSIS-LOO scores see Tables 1, S2, S3; for a more detailed description of the model space see Supplementary Methods). BMS resulted in a protected exceedance probability of *pxp* = 0.998 (excluding flawed PSIS-LOOs: *pxp*_*LOOcorr*_ > 0.999) for Model 6 and a *BOR* < 0.001 (excluding flawed PSIS-LOOs: *BOR*_*LOOcorr*_ < 0.001).

**Table 1:** Model comparisons.

Model	PSIS-LOO	LOO-SE	LOO-Diff (SE-Diff)	% of $\hat{k}$ > 0.7	No. Est. Parameters
**Agent-LOOP (Experiment 1 and 3)**					
Self = Other					
Unity Model (M1)	−2380.1	247.8	135.4 (63.7)	0.1	5
Ability Model (M2)	−2336.5	261.5	91.7 (42.4)	0.3	6
Valence Model (M3)	−2320.5	259.0	75.7 (49.4)	0.2	6
Self ≠ Other					
Unity Model (M4)	−2376.2	254.8	131.5 (54.6)	0.4	6
Ability Model (M5)	−2330.7	263.3	85.9 (42.8)	1.2	8
Valence Model (M6)	−2244.8	283.5	—	0.3	8
Mean Model (M7)	−2953.6	190.3	708.9 (123.3)	0.0	4
**Audience-LOOP (Experiment 2)**					
Unity Model (M1)	−708.2	145.1	213.1 (35.8)	0.1	3
Ability Model (M2)	−570.2	150.0	75.0 (26.8)	0.3	4
Valence Model (M3)	−495.2	150.9	—	0.1	4
Mean Model (M4)	−1189.5	124.9	694.4 (61.3)	0.0	2

*Note*. LOO = sum PSIS-LOO, approximate leave-one-out cross-validation (LOO) using Pareto-smoothed importance sampling (PSIS); LOO-SE = Standard error of PSIS-LOO; LOO-Diff (SE-Diff) = Difference in expected predictive accuracy (PSIS-LOO) for all models from the model with the highest PSIS-LOO (Valence Model) and standard errors of differences; percentage of $\hat{k}$ *-* estimated shape parameters of the generalized Pareto distribution - exceeding 0.7 (all according to Vehtari *et al*.^70^); No. Est. Parameters = number of estimated parameters in the model.

For the Audience-LOOP (experiment 2) there was a clear indication that the Valence Model (Model 3) outperformed all other models according to BMS. Across the Private and Public groups, the protected exceedance probability for the Valence Model was *pxp > *0.999 (*pxp*_*LOOcorr*_* > *0.999). The BOR was *BOR* < 0.001 (*BOR*_*LOOcorr*_ < 0.001).

Taking into account that model comparisons consistently favored the Valence Model across experiments (Model 6 for the Agent-LOOP and Model 3 for the Audience-LOOP) the Valence Model was selected for all further analyses of learning parameters. Model selection thus revealed that a learning model far surpasses a mean model without learning, and that amongst the learning models, those assuming different learning rates for positive and negative PEs performed best, confirming that it is important to distinguish how positive and negative information is processed. This allowed us to specifically test our main hypotheses of difference in learning about the self vs the other with respect to negative in contrast to positive PEs.

The time courses of EXP ratings predicted by our winning model successfully captured trial-by-trial changes in EXP due to PE updates within each of the ability conditions at the individual subject level (*R*^*2*^ = 0.37 ± 0.24; *M* ± *SD*) supporting the validity of the model in describing the subjects’ learning behavior. Posterior predictive checks also confirmed that the winning model captured the core effects in our model free analysis by showing that behavioral analysis on the predictions recapitulates the tendency towards more negative performance expectations for the other that was core to our data (see Supplementary Results and Fig. 2).

### Learning parameters

#### Experiment 1: Agent-LOOP

**Figure 4:**
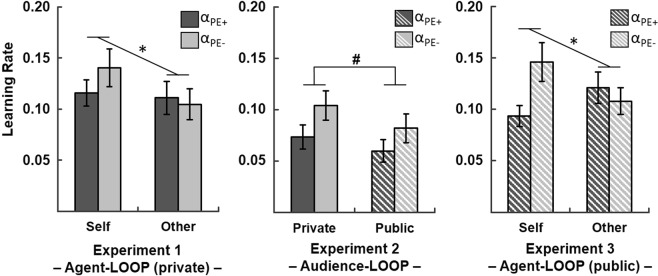
Learning rates across the three experiments. The learning rates derived from the Valence Model indicate that there was a bias towards increased updating in response to negative prediction errors (α_PE−_) in contrast to positive prediction errors (α_PE+_) across all three experiments. This effect was only present when learning about the self (see left and right) and independent of the social context. Bars represent mean learning rates, error bars depict +/− 1 standard error; *indicates a significant interaction effect of PE Valence ^x^Agent; ^#^indicates a significant main effect of PE Valence across Audience groups.

Participants showed higher learning rates when learning about themselves compared to learning about another person (main effect of Agent: *F*_*(1,22)*_ = 5.23, *p* = 0.032). There was no main effect of PE Valence (*F*_*(1,22)*_ = 0.90, *p* = 0.354), but the significant interaction of Agent × PE Valence (*F*_*(1,22)*_ = 5.49, *p* = 0.029) suggested that there was a bias of updating towards negative information when learning about the self (*t*_*(22)*_ = 1.79, *p* = 0.088, *M*(α_PE−(S)_) = 0.14, *SD* = 0.09; *M*(α_PE+(S)_) = 0.12, *SD* = 0.06). Learning about another person’s performance did not show a significant bias towards negative valence (*t*_*(22)*_ = −0.71, *p* = 0.484; *M*(α_PE−(O)_) = 0.10, *SD* = 0.07; *M*(α_PE+(O)_) = 0.11, *SD* = 0.08; see Fig. 4).

#### Experiment 2: Audience-LOOP

The results of the Audience-LOOP replicated the updating bias towards negative self-related information (main effect of PE Valence: *F*_*(1,57)*_ = 12.64, *p = *0.001; Private: *M*(α_PE−_) = 0.10, *SD* = 0.08, *M*(α_PE+_) = 0.07, *SD* = 0.06; Public: *M*(α_PE−_) = 0.08, *SD* = 0.08, *M*(α_PE+_) = 0.06, *SD* = 0.06). We, however, did not find any differences in learning rates between the Private and the Public group (*F*_*(1,57)*_ = 1.14, *p* = 0.290), nor a significant interaction of Audience × PE Valence (*F*_*(1,57)*_ = 0.35, *p* = 0.559), suggesting that being in public might not affect the level of updating in response to negative or positive information per se.

#### Experiment 3: replication and extension

We again found a significant interaction of Agent × PE Valence (*F*_*(1,28)*_ = 15.45, *p* = 0.001), replicating our previous findings of a bias towards negative information, when learning about the self (*t*_*(28)*_ = 3.57, *p* = 0.001; *M*(α_PE−(S)_) = 0.15, *SD* = 0.10; *M*(α_PE+(S)_) = 0.09, *SD* = 0.05) and no bias towards negative valence when learning about the other person (*t*_*(28)*_ = −1.35, *p* = 0.132; *M*(α_PE−(O)_) = 0.11, *SD* = 0.07; *M*(α_PE+(O)_) = 0.12, *SD* = 0.08). Unlike in experiment 1 learning rates did not differ between the Self and the Other condition (*F*_*(1,28)*_ = 0.13, *p* = 0.718), due to slightly increased learning rates for the Other condition in the Public group. The main effect of PE Valence reached significance (*F*_*(1,28)*_ = 5.25, *p* = 0.030), but was driven by a strong bias towards negative valence only in the Self condition. Considering the estimated learning rates of the private Agent-LOOP in experiment 1 and the public version in experiment 3 for the assessment of audience effects, the main effect of PE Valence (*F*_*(1,50)*_ = 4.99, *p* = 0.030) as well as the interaction of Agent × PE Valence (*F*_*(1,50)*_ = 19.01, *p* < 0.001) remained significant, while the main effect of Agent still failed to reach significance (*F*_*(1,50)*_ = 1.98, *p* = 0.166). Interestingly, replicating the results of the Audience-LOOP we could not find a main effect of Audience (Audience: *F*_*(1,50)*_ < 0.01, *p* = 0.966) or any interaction effects (Audience × Agent: *F*_*(1,50)*_ = 0.67, *p* = 0.416; Audience × PE Valence: *F*_*(1,50)*_ = 0.68, *p* = 0.414; threefold-interaction Audience × PE Valence × Agent: *F*_*(1,50)*_ = 2.44, *p* = 0.125). This again suggests that the presence of an audience might not affect updating in response to negative or positive information per se.

Finally, cumulative Bayesian analysis suggests that across all three experiments there was extremely high evidence^43^ for a negative valence bias (Bayes Factor_10_ = 19081.7; effect size δ = −0.68, 95%-confidence interval (CI) = [−0.41/−0.95]). Even when adopting an informed prior in favor of a positivity bias (medium mean effect size = 0.5; standard deviation = 0.25), as has been suggested by various studies^13^, there still was very strong support for a negativity bias in our data (Bayes Factor_10_ = 94.4; effect size δ = −0.36, CI = [−0.18/−0.55]).

#### Associations of learning behavior with self-esteem and social anxiety

Partial correlations of Valence Bias Scores (Valence Bias Score = (α_PE+(S)_ − α_PE−(S)_)/(α_PE+(S)_ + α_PE−(S)_); similarly for other-related learning)^44,45^ and EXP ratings indicated that Valence Bias Scores successfully captured behavioral variance between individuals for all three experiments: Agent-LOOP (experiment 1): *r*_*part*_ = 0.71, *p* < 0.001, Audience-LOOP: Private: *r*_*part*_ = 0.78, *p* < 0.001, Public: *r*_*part*_ = 0.86, *p* < 0.001, Agent-LOOP (experiment 3): *r*_*part*_ = 0.47, *p* = 0.006. Thus, individuals with more negative Valence Bias Score ended up with lower self-related performance expectation in the end of the task (controlled for the initial expectations).

**Figure 5:**
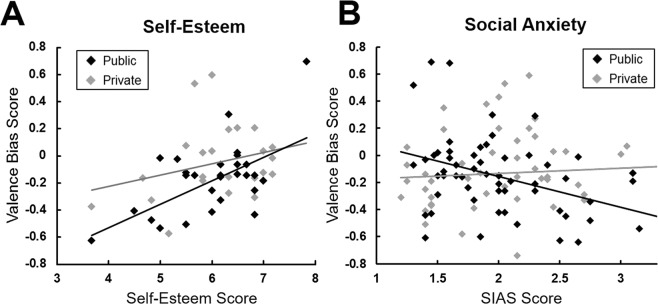
Correlation plots of self-related Valence Bias Scores and social anxiety as well as self-esteem for the public and private groups. (**A)** Increased trait self-esteem (SDQ-III score) was associated with a decrease in the negative updating bias about the self in the Public (experiment 3) and the Private group (experiment 1). (**B)** Trait social anxiety (SIAS score) was associated with increased self-related learning biases towards negative information in the Public groups but not the Private groups (across all experiments).

The valence bias in self-related learning we found across all three experiments (Valence Bias Score) was negatively associated with interindividual differences in self-esteem in the Agent-LOOP task, *r*_*(52)*_ = 0.44, *p = *0.001 (across experiment 1 and 3; see Fig. 5A). This indicates that individuals with lower self-esteem showed a stronger valence bias in learning from negative PEs compared to positive PEs. Bayesian analysis corroborated this finding and showed strong evidence for an association of self-esteem and Valence Bias Score (Bayes Factor_10_ = 30.0; effect size δ = 0.44, CI = [0.18/0.63]) but the data was inconclusive with regard to a modulating effect of Audience (Bayes Factor_10_ = 0.6).

When assessing the impact of trait social anxiety on updating in response to negative vs positive PEs, we found that the Valence Bias Score was significantly negatively associated with SIAS scores in the Public groups (*r*_*(58)*_ = −0.39, *p* = 0.002; across experiment 2 and 3), while there was no association in the Private groups (*r*_*(50)*_ = 0.06, *p* = 0.669; across experiment 1 and 2; difference of correlations: z = 2.39, *p* = 0.018). This indicates that individuals higher in social interaction anxiety shifted their updating behavior more strongly towards learning from negative information, specifically when they were in a public context. This association of the SIAS with learning biases in the public but not in the private context was not present for learning rates for the other person’s performance (Private: *r*_*(23)*_ = −0.03, *p* = 0.897; Public *r*_*(29)*_ = −0.21, *p* = 0.263; difference in correlations: *z* = 0.64, *p* = 0.524). Bayesian analyses revealed moderate support that the association of SIAS scores and self-related Valence Bias Scores was modulated by the Audience (Bayes Factor_10_ = 7.2). We could find strong evidence that SIAS scores were negatively associated with the Valence Bias in the Public group (Bayes Factor_10_ = 14.4; effect size δ = −0.39, CI = [−0.14/−0.58]), while there was support for the absence of that effect in the Private group (Bayes Factor_10_ = 0.2; effect size δ = 0.06, CI = [−0.21/0.32]).

## Discussion

In a series of three consecutive experiments we explored how individuals update beliefs about their own abilities, and contrasted this against how they update beliefs about others. We aimed to disentangle situational, motivational, and interindividual factors to better understand the nature of learning biases and their relevance for the development of self-concepts. With regard to our first main question, we found that individuals show an updating bias towards negative information about their own performances. When people witnessed feedback about the performance of others, they were as sensitive to positive as negative information. When finding out about their own performance they learnt most from negative feedback, which updated their performance estimates more than the positive feedback.

The self-related negativity bias in our findings stands in opposition to the view that self-related learning is positively biased in general^13^ and even when adopting a biased prior in favor of a positivity bias, our study provides clear evidence for a negativity-bias in the LOOP task. Hence, one could argue that these results should motivate a closer look on the specific features of tasks applied to examine biases in self-related learning. We argue that several features of the LOOP task introduced here differentiate our task from those that provide evidence in favor of a general positivity bias^15–17,46^.

The specificity of findings on self-related learning suggest that when individuals learn about their own abilities, in contrast to learning about another person, unique motivational factors come into play and shape the way of thinking about and learning from self-related feedback. Subjective desirability of information is considered a constituting factor leading to a positivity bias^13^. Such biased updating is in line with the common phenomenon of overconfidence or the so-called “better-than-average” effect, describing the phenomenon that people tend to judge their own performance as better than the average performance^47,48^. While individuals are typically inclined to hold a positive view of themselves^49^, which can shape the abovementioned situational desirability of positive information, the nature of the performance situation determines how individuals achieve a positive view of the self in the long run. Depending on the situation, two distinct and almost oppositional motives could in principle color how we process feedback: self-enhancement (i.e. the tendency to evaluate the self positively by either augmenting the positivity or diminishing the negativity of the self-concept) and self-improvement (i.e. the tendency to improve one’s own performance to maintain a positive self-evaluation)^20,49^.

Studies typically find a positivity bias in updating in line with self-enhancement motives when individuals are confronted with feedback in a personally relevant domain (e.g. IQ, health status), but the content (e.g. being intelligent or not) is rather unchangeable during the experiment^15,16,18,46^. Here, self-enhancement motives are triggered because a negative self-related belief in the personally relevant domain would pose a threat for the individual^20,49^. Being unable to change the actual outcome with regards to the self, self-enhancement remains the only behavioral option to fulfill the wish for a positive self-view^20^ and thus might increase the motivation for a positive updating bias when confronted with new information about the self^16,23^.

In contrast, in our task we explicitly aimed to induce a state of experienced control over the outcome of the situation – by making participants believe they received online feedback on their actual task performance – and thus likely triggered participants’ self-improvement motives^20,50^. A situation that allows for improvement is thought to naturally trigger people’s desire for self-improvement specifically in response to past failure^51^ and when upward social comparison information is provided^50^. Given the participants feel they have the necessary psychological resources (i.e. sufficient self-esteem, low self-threat induced by a novel task), they should focus on the negative feedback – here trials that, in particular, offer room for improvement – and be motivated to do better in the current performance situation^50^. The increased significance of negative feedback, that has also been shown to shape performance forecasts^52,53^, might have led to the biased updating behavior we find in the current study. This particular study set-up aligns nicely with many real-life performance situations at school or work environments, in which negative feedback calls for direct behavioral change^50^. One needs to consider, that this interpretation is speculative in the context of our experiments and we do not know if the motivation to improve might have driven the individuals’ learning behavior. While participants did believe that the relative performance feedback was related to their actual task performance, most likely believing they could change the next trial’s outcome with their behavior, participants did not receive concrete feedback on their estimation accuracy and were not actually able to improve their performance. An alternative factor shaping learning biases might be the reduced relevance of the estimation task in contrast to learning about one’s IQ or health risks, which might have reduced positivity biases.

An additional explanation for the negativity bias might be grounded in affective processes associated with negative feedback. From earlier research we know that failing unexpectedly in a performance situation not only triggers the motivation to improve in the next trial, but can also elicit the experience of embarrassment (even by the mere thought or possibility of an audience witnessing one’s mistakes) and might induce a fear of failing again^33,54^. Such an affective connotation of negative prediction errors might thus similarly increase the subjective relevance of negative information resulting in a more negatively biased learning as it is thought to form self-efficacy beliefs^1^. Future studies will be needed to directly test the impact of distinctive affective states like embarrassment and motives of self-enhancement or improvement as well as task-related effects on specific biases in self-related learning.

With our second main question we assessed the impact of interindividual personality differences on self-related belief formation. Apart from a general updating bias towards negative information about the self we observed that the asymmetry in learning rates was associated with prior beliefs about the self. Individuals with more negative prior beliefs about themselves (i.e. lower self-esteem) showed more pronounced learning biases towards negative information suggesting that, besides a general valence induced bias, confirmation biases shape social learning processes. The term “confirmation bias” describes the observation that one favors information in line with one’s prior views and it is argued that the confirmation bias might be especially pronounced with regards to self-concepts^23^. Such an impact of prior beliefs is supported by previous studies showing that individuals preferably updated their beliefs in line with their prior expectations about their own task performance^53^. Similarly, low self-esteem has been shown to lead individuals to confirm their prior beliefs, maintaining negative performance expectations even in the context of successful performance^22^. In this line, interindividual differences in updating behavior have been associated with distinct activation patterns on the neural systems level. For example, trait optimism has been associated with decreased activation of the right inferior prefrontal gyrus in response to negative self-related information, indicating decreased sensitivity for negative information that is incongruent with more pronounced optimistic beliefs^15^.

Apart from self-esteem, trait social anxiety is a potent modulator of biases in self-related learning. In the present studies we directly addressed the fundamental fear in social anxiety: being observed in an evaluative performance situation. In line with recent findings, individuals higher in social anxiety exhibited an increased bias towards negative information^39,40^. Interestingly, with regard to our third main question, this learning bias towards negative information was modulated by the social context and only present when participants were exposed to a potentially judging audience. This distinction has not been explored so far in social anxiety even though the importance of the social context has recently been pointed out for depression^55^. It has been suggested that diminished striatal involvement in the brain’s reward system reflects the lack of a motivational preference for positive social information in social anxiety disorder^56^. However, given our data we would question whether such a valence bias in social anxiety exists independently of the social context. Previous studies reported that socially anxious individuals displayed negativity biases in response to social evaluative feedback on a public speech^39^ or social feedback in the form of “personal-descriptive adjectives”^40^, information that is likely to trigger social fear related thought and attention patterns. This is corroborated by studies suggesting that individuals suffering from social anxiety typically pay more attention to information indicating a potential threat to their social image and interpret these social cues in a negatively biased way^29,57–60^. In a previous study, we demonstrated that socially anxious individuals paid increased attention to the audience while receiving feedback on their estimation performance. Also, pupil dwell time on the faces of the audience mediated neural activation differences in the mentalizing network^33^. Taken together, one may assume that the informational content (i.e. social evaluative feedback) as well as context (i.e. publicity or privacy) modulate attentional and cognitive processes in social anxiety. Here, we explicitly considered the impact of content and context and designed our task to be generally unrelated to social or other specific fears (unlike other commonly employed tasks in social learning where one e.g. is being judged by others with regards to one’s personality). Thus, interindividual differences in updating behavior in the present data likely reflect a more basic bias in self-related information processing compared to tasks which comprise strong priors (e.g. “I have always been awkward in social interactions.”)^39,40^. Our finding of context specific biases in self-related learning thus provides first indications that biased self-related belief updating only emerges when fear related processes are triggered, i.e. when confronting (socially anxious) individuals with potential social judgement by an evaluative audience. However, as our sample consisted of healthy individuals with non-clinical levels of social anxiety, future studies on clinical populations are needed to substantiate this clinically relevant claim.

When taking self-improvement motives into account, increased responsiveness to negative feedback related to high levels of social anxiety or low self-esteem might be a strategy to make up for perceived personal deficits. However, it has been shown that people low in self-esteem rather show a decline in task performance instead of benefitting from negative feedback^24^, while high self-esteem is thought to facilitate task persistence^61^. Similarly, instead of improving their social performance, socially anxious individuals feel unable to make the desired positive impression, which in turn increases the experience of social anxiety^62^ and the kind of avoidance behavior that contributes to the often described deficits in everyday life functioning in social anxiety disorder^29^. Self-related confirmation biases in learning might thus, at least in some cases, confirm maladaptive and rather pessimistic views about the self – or “more realistic”, as discussed in the context of depression^25,63^. With respect to social anxiety this might reflect a core process of clinical relevance that could explain the persistence of negative self-related beliefs in the social domain that is not necessarily grounded in actual negative feedback^29^. In line with this, another explanation for the pronounced negativity bias in the current experiments might be that individuals have overly negative prior self-related beliefs for their estimation ability, which could lead to a confirmation bias for their own inability to solve the estimation questions. Identifying the circumstances under which a preference for negative feedback might be triggered and those that lead to self-improvement vs. consistent negative beliefs, are two interesting avenues for future research.

To summarize, our results indicate a negativity bias when forming beliefs about one’s own abilities in a performance situation that is shaped by prior beliefs about the self (self-esteem) in line with a confirmation bias. With the current task we were able to form people’s beliefs about their own abilities within a short period of time. Such beliefs are often considered as rather stable and form the basis for human behavior in everyday social and professional life. Being able to induce and observe self-related learning processes enables us to further disentangle the basic mechanisms underlying the persistence of (negative) self-images as well as potential behavioral consequences specifically in individuals low in self-esteem or high in social anxiety. Thus, the present findings are of high relevance for developmental, educational or clinical applications.

The LOOP task introduced here has a number of unique features that we believe are important to illuminate a wider gamut of learning situations. While past research had focused on feedback on highly valued and difficult to change aspects of the self, our task explores learning in changeable and relatively neutral domains. The online performance-feedback loop suggests that people have an opportunity to directly use feedback to improve performance and the novel and neutral content of the task reduces the impact of domain specific prior beliefs. We are curious if the observed negativity bias would hold over a variety of self-related performance tasks that suggest an immediate opportunity to improve. We believe that by challenging the generalizability of the positivity bias the current study points to the importance of situational, motivational, and interindividual factors in self-related belief formation. While overly negative distortions of self-related learning might have far-reaching consequences for decisions that are crucial for everyday life our finding might also encourage a discussion about the value of recognizing personal failures as a prerequisite for improvement. Taking into account that much of what people believe is biased or even wrong^64^, such intellectual humility^65^ to focus on one’s shortcoming or even “stupidity”^66^ have recently been coined as key components for progress in research and likely a lot of other areas of life.

## Materials and Methods

### Participants

The study was approved by the ethics committee of the University of Lübeck (AZ 16-315, AZ 17-220), has been conducted in compliance with the ethical guidelines of the American Psychological Association (APA), and all subjects gave written informed consent. All participants were recruited at the University Campus of Lübeck, were fluent in German, and had normal or corrected-to-normal vision. All participants received monetary compensation for their participation in the study. Across all three experiments seven subjects were excluded after participation because they did not believe the cover-story of the task. For the first experiment we initially recruited 26 participants and included 24 (12 female, aged 20–31 years; *M* = 23.75; *SD* = 3.22). For the second experiment we initially recruited 64 subjects and included 61, who were randomly assigned to either a Private or a Public social context group. The Private group consisted of 30 participants (20 female, aged 18–32 years; *M* = 22.27; *SD* = 3.01), the Public group of 31 participants (22 female, aged 19–32 years; *M* = 22.58; *SD* = 2.69). For the third experiment we initially recruited 32 participants and included 30 (24 female, aged 18–30 years; *M* = 21.70; *SD* = 3.33). For details on the sample characteristics see Supplementary Table S1.

### General procedure

#### Learning of own performance task

The Learning of own performance (LOOP) task enables participants to incrementally learn about themselves from trial-by-trial performance feedback in a task testing their own abilities. For this purpose we adapted a cognitive estimation task that we implemented in a previous study on the induction of embarrassment^33^. For the LOOP task all participants were invited to take part in an experiment on “cognitive estimation”. Participants needed to estimate properties of different objects (e.g. the height of houses or the weight of animals). To make participants learn about their estimation ability, they received manipulated performance feedback in two distinct estimation categories. Unbeknownst to the participant, one category was arbitrarily paired with High Ability and one with Low Ability feedback (e.g. “height” of houses = High Ability and “weight” of animals = Low Ability or vice versa; estimation categories were counterbalanced between Ability conditions) independently of the actual responses given by the participants. Thus, participants could learn over the course of the experiment that they performed well in one estimation category and poorly in the other. Introducing a High and a Low Ability condition also increased the variance of positive and negative prediction errors (PEs) and allowed us to assess PE valence specific effects. Performance feedback was provided after every estimation trial during the task so that participants could use the last feedback in order to adapt their predictions of the performance feedback for the next trial of the same condition. Importantly, by implementing a continuous performance-feedback-loop participants were made to believe that they could utilize the feedback in order to improve their cognitive estimation performance, e.g. to increase their efforts following negative feedback. Fixed performance feedback sequences were presented for all participants, indicating their current estimation accuracy as percentiles compared to an alleged reference group of 350 university students who, according to the cover-story, had been tested beforehand (e.g. “You are better than 96% of the reference participants.“; see Fig. 1A). Participants never received feedback on how close their actual performance was to the “correct” answer. Presenting estimation accuracies by means of percentiles therefore ensured that participants were more likely to believe that the feedback represented their actual performance. In the Low Ability condition, feedback was approximately normally distributed around the 35th percentile (SD ≈ 16; range 1–60%) and in the High Ability condition around the 65th percentile (SD ≈ 16; range 40–99%).

In the beginning of each trial a cue (CUE) was presented indicating the estimation category (e.g. “height”, which could correspond to the High Ability condition) and participants were asked to indicate their expected performance (EXP) for this trial on the same percentile scale used for feedback. Participants were told accurate EXP ratings would be rewarded with up to 6 cents per trial, i.e. the better their EXP rating matched their actual feedback percentile the more money they would receive, to increase motivation and encourage honest response behavior. Following each EXP rating, the estimation question was presented for 10 seconds (EST). During the EST period, continuous response scales below the pictures determined a range of plausible answers for each question, and participants indicated their responses by navigating a pointer on the response scale with a computer mouse. Subsequently, feedback (FB) was presented for 5 seconds (see Fig. 1A). All stimuli were presented using MATLAB Release 2015b (The MathWorks, Inc.) and the Psychophysics Toolbox^67^. The LOOP formed the main frame for all three experiments. The adaptations of the LOOP for each experiment are explained below.

#### Experiment 1: Agent-LOOP

For the Agent-LOOP two participants were invited at the same time. Participants were informed they would take turns with the other participant, either performing the task themselves (Self) or observing the other person performing (Other). In the beginning of each trial the CUE indicated who’s turn it was (e.g. “Thomas” or “You”) along with the estimation category depicted below (e.g. “height”; estimation categories were counterbalanced between Ability conditions and Agent conditions (Self vs. Other)). Depending on the corresponding condition participants then indicated their EXP rating either for their own or the other participant’s performance. At the end of each trial, performance feedback was always presented to both participants. Participants thus underwent four feedback conditions with 25 trials each (Agent condition (Self vs Other) × Ability condition (High Ability vs Low Ability)). Trials of all conditions were intermixed in a fixed order with a maximum of two consecutive trials of the same condition.

#### Experiment 2: Audience-LOOP

In experiment 2 we implemented another version of the LOOP task to assess the impact of the presence of an audience on self-related learning in a between-subject design (Fig. 1C). Participants were invited alone and randomly assigned to one of two experimental groups. In the Private group participants completed the estimation task as described above all on their own. In the Public group the experimenter, who represented the audience, was seated behind the participant and observed his/her performance, allegedly in order to assess additional performance characteristics that could not be recorded by the computer. The following part of the experiment including the estimation task was executed as described above. For each of the two self-related Ability conditions (High Ability vs Low Ability) 30 trials were presented intermixed in a fixed order with a maximum of two consecutive trials of the same condition.

#### Experiment 3: replication and extension

In experiment 3 we used the Agent-LOOP task (experiment 1) and additionally introduced publicity in a more minimal fashion compared to the Audience-LOOP (experiment 2). To do so, instead of seating someone behind the participants, we simply manipulated the amount of information participants were able to see from each other. Thus, all participants were told that they were randomly selected for the Public group by the computer (i.e. being observed), while allegedly the other participant was in the Private condition (i.e. being the observer). Like in the Agent-LOOP in experiment 1, participants were only able to see the other participant’s performance feedback, but were told that their EXP ratings were made public for the other participant. This minimal change in the paradigm was expected to make participants experience being observed by and exposed to the other’s judgement, while at the same time being unable to observe and judge the other person equally. This was confirmed by our debriefing questionnaire indicating that only 9% of participants in the private Agent-LOOP were bothered by the other participant observing their performance while in the public version 38% reported the same.

### Statistical analysis

#### Model free behavioral analysis

A model free analysis was performed on the participants’ EXP ratings for each trial to illustrate the basic effects we see in our behavioral data. For the Agent-LOOP task (experiments 1 and 3) a repeated-measures ANOVA was calculated with the factors Trial (25 Trials) × Ability condition (High Ability vs Low Ability) × Agent condition (Self vs Other). For the Audience-LOOP (experiment 2) we calculated a repeated-measures ANOVA with the factors Trial (30 Trials) × Ability condition (High Ability vs Low Ability) and Audience (Public vs Private) as a between subject factor. Additionally, we collapsed the data of experiments 1 and 3 to replicate and extend the conclusions on the impact of the audience on learning about the self and another person. The corresponding ANOVA included Audience (Public vs Private) as an additional between-subject factor. After model fitting four subjects had to be excluded from further analyses (see section 2.4.3). To keep the sample consistent across analyses, model free behavioral analyses were also conducted on the reduced sample and results remained consistent with those computed on the full sample (see Supplementary Results).

#### Computational modeling of learning behavior

We modeled dynamic changes in self-related beliefs for all EXP ratings participants provided in the beginning of each trial in response to the provided performance FB using prediction error delta-rule update equations (adapted Rescorla-Wagner model; see Fig. 1B)^68^. The model space is described in the Results section and depicted in Fig. 3. In our task, Ability condition and PE valence were correlated in the sense that the Low Ability condition contained more negative PEs and the High Ability condition more positive PEs, assuming that participants initially expect their own performance to be around the 50^th^ percentile. Nevertheless, if the Valence Model won it could be assumed that PE valence is the more prominent factor affecting learning rates compared to the Ability condition and vice versa.

In addition to the learning rates we either fitted parameters for the initial belief about the own and the other participant’s performance, separately or combined for both ability conditions, or used the initial performance expectation ratings as fixed starting values. The models presented in the Results section included initial belief parameters for each condition separately (see Supplementary Methods for a detailed description of the complete model space).

#### Model fitting

For model fitting we used the RStan package (Stan Development Team, 2016. RStan: the R interface to Stan. R package version 2.14.1.), which uses Markov chain Monte Carlo (MCMC) sampling algorithms. All of the learning models in the model space were fitted for each subject in the corresponding experimental group. Posterior parameter distributions were sampled for each subject. A total of 2400 samples were drawn after 1000 burn-in samples (overall 3400 samples; thinned with a factor of 3) in three MCMC chains. We assessed if MCMC chains converged to the target distributions by inspecting $\hat{R}$ values for all model parameters^69^. Three subjects (n = 1 for each of the experiments) were excluded because at least one model parameter had $\hat{R}$ values exceeding 1.1 indicating non-convergence of the MCMC chains, which was confirmed by visual inspection. An additional subject was excluded after visual inspection due to implausible model parameters, i.e. mean learning rate of 1, which was more than 10 standard deviations above average (experiment 2). Effective sample sizes (*n*_*eff*_) of model parameters, which are estimates of the effective number of independent draws from the posterior distribution, were typically greater than 1000 (>1300 for most parameters). Posterior distributions for all parameters for each of the subjects were summarized by their mean as the central tendency resulting in a single parameter value per subject that we used in order to calculate group statistics. Using the median lead to similar conclusions.

#### Bayesian model selection and family inference

In order to select the model that most likely guided the participants’ updating behavior, as a first step, we estimated pointwise out-of-sample prediction accuracy for all fitted models separately for each participant by approximating leave-one-out cross-validation (LOO; i.e. corresponding to leave-one-trail-out per subject) as recommended for assessing model fit without introducing penalties for model complexity^70,71^. To do so we applied Pareto-smoothed importance sampling (PSIS) using the log-likelihood calculated from the posterior simulations of the parameter values as implemented by Vehtari *et al*.^72^. Sum PSIS-LOO scores for each model as well as information about $\hat{k}$ values – the estimated shape parameters of the generalized Pareto distribution – indicating the reliability of the PSIS-LOO estimate are depicted in Table 1. As summarized in Table 1 very few trials resulted in insufficient parameter values for $\hat{k}$ and thus potentially unreliable PSIS-LOO scores (on average 0.17 trials per subject with $\hat{k}$ > 0.7)^72^. Visual inspection of the corresponding subjects suggested that in some cases subjects had provided EXP ratings far away from the current average, PSIS-LOO scores for the corresponding trials were, however, mostly within the range of the other trials. In order to make sure that these trials would not bias the model selection processes, we excluded the PSIS-LOO scores for these trials and repeated the model selection procedure replicating our model selection results. Bayesian model selection (BMS) on PSIS-LOO scores was performed on the group level accounting for group heterogeneity in the model that best describes learning behavior^73^. This procedure provides the protected exceedance probability for each model (*pxp*), indicating how likely a given model has a higher probability explaining the data than all other models in the comparison set, as well as the Bayesian omnibus risk (*BOR*), the posterior probability that model frequencies for all models are all equal to each other^73^. We also provide difference scores of PSIS-LOO in contrast to the model that won the BMS that can be interpreted as a simple ‘fixed-effect’ model comparison (see Tables 1 and S2, S3)^71,72^. Mostly, model comparisons according to PSIS-LOO difference scores were qualitatively comparable to the BMS analyses for our data.

#### Posterior predictive checks and statistical analyses of learning parameters

First, posterior predictive checks were conducted by quantifying if the predicted data could capture the variance in EXP ratings for each subject within each of the experimental conditions using Regression analyses. Additionally, we repeated the model free analysis we had done on the behavioral data with the data predicted by the winning model to assess if the winning model captured the core effects in the behavioral data. Additionally, correlations between the parameters within the winning model were assessed (see Supplementary Results and Supplementary Tables S4–S6).

Model parameters, i.e. learning rates, of the winning models for all experiments were analyzed on the group level using IBM SPSS Statistics for Windows, Version 22.0 (IBM Corp., 2013, Armonk, NY). For the Agent-LOOP in experiment 1 a repeated-measures ANOVA was calculated on the learning rates with the factor Agent (Self [α_PE+(S)_, α_PE−(S)_] vs Other [α_PE+(S)_, α_PE−(S)_]) and factor PE Valence (PE+ [α_PE+(S)_, α_PE+(O)_] vs PE− [α_PE−(S)_, α_PE−(O)_]) testing if negative feedback gains a specific weight when learning about the self vs the other.

In the Audience-LOOP we assessed the impact of the social context, i.e. the presence of an evaluative audience, on self-related belief updating and its interaction with social anxiety. Here, an ANOVA was implemented with PE Valence (PE+ [α_PE+_] vs PE− [α_PE−_]) as a within-subject factor and Audience (Public vs Private) as a between subject factor.

For experiment 3, as for the Agent-LOOP in experiment 1, a repeated-measures ANOVA was calculated on the learning rates with the factors Agent and PE Valence. Additionally, we collapsed the learning rates of experiments 1 and 3 to directly test the impact of the audience on learning about the self and another person. We thus implemented another ANOVA including Audience (Public vs Private) as an additional between-subject factor.

To investigate the associations of learning biases with the subjective prior sense of self-esteem, i.e. SDQ-III scores (available for experiment 1 and 3), as well as social anxiety, i.e. SIAS scores, we calculated a normalized learning rate Valence Bias Score for self-related learning (Valence Bias Score = (α_PE+(S)_ − α_PE−(S)_)/(α_PE+(S)_ + α_PE−(S)_)) and similarly for other-related learning^44,45^. Pearson correlations were calculated between Valence Bias Score and personality traits. Context specific effects of social interaction anxiety on self- vs other-related updating behavior were assessed by contrasting correlations between the public and private groups. For all three experiments we additionally tested if the Valence Bias Score was suitable to capture interindividual differences in how subjects changed their beliefs about themselves over time by calculating partial correlations between Valence Bias Scores and the average of the last two EXP ratings for both ability conditions controlling for the average of the first two EXP ratings for both ability conditions.

Finally, cumulative Bayesian analyses (using JASP Version 0.9, ASP Team, 2018) were implemented collapsing the data for all experiments in order to assess the overall evidence for self-related learning biases as well as associations of such learning biases with social anxiety and self-esteem. Here, we first assessed the impact of the audience on each of the effects. A Bayesian ANOVA with the factors PE Valence and Audience was thus calculated on the learning rates and Bayesian linear regressions of personality traits and Valence Bias Scores were calculated including the factor Audience and the interaction of Audience and personality traits. Effect sizes for self-related learning biases were then calculated using Bayesian t-tests on the learning rates and Bayesian correlations were calculated to assess the associations of learning biases with personality traits. In case there was evidence for an audience effect, effect sizes were calculated separately for the Public and the Private group.

## Supplementary information

Supplementary Information

## Data Availability

The datasets generated and analyzed during the current study are available from the corresponding author on reasonable request.
