# Supplementary material for: Negativity-bias in forming beliefs about own abilities
Source: Sci Rep. 2019 Oct 8;9:14416. doi: 10.1038/s41598-019-50821-w (PMC6783436; doi:10.1038/s41598-019-50821-w)
Supplement: Supplementary file 1 — Supplementary Information [file 41598_2019_50821_MOESM1_ESM.docx]

**Title:** Negativity-bias in forming beliefs about own abilities

**Authors:** Laura Müller-Pinzler1,2, Nora Czekalla1, Annalina V Mayer1, David S Stolz1, Valeria Gazzola2,3, Christian Keysers2,3, Frieder M Paulus1, Sören Krach1

**Email:**

Laura Müller-Pinzler* [mueller-pinzler@snl.uni-luebeck.de](mailto:mueller-pinzler@snl.uni-luebeck.de)

Nora Czekalla [czekalla@snl.uni-luebeck.de](mailto:czekalla@snl.uni-luebeck.de)

Annalina V Mayer [mayer@snl.uni-luebeck.de](mailto:mayer@snl.uni-luebeck.de)

David S Stolz [stolz@snl.uni-luebeck.de](mailto:stolz@snl.uni-luebeck.de)

Valeria Gazzola [v.gazzola@nin.knaw.nl](mailto:v.gazzola@nin.knaw.nl)

Christian Keysers [c.keysers@nin.knaw.nl](mailto:c.keysers@nin.knaw.nl)

Frieder M Paulus [paulus@snl.uni-luebeck.de](mailto:paulus@snl.uni-luebeck.de)

Sören Krach [krach@snl.uni-luebeck.de](mailto:krach@snl.uni-luebeck.de)

**Affiliations:**

1: Department of Psychiatry and Psychotherapy, Social Neuroscience Lab, University of Lübeck, Ratzeburger Allee 160, D-23538 Lübeck, Germany

2: Social Brain Lab, Netherlands Institute for Neuroscience, KNAW, Meibergdreef 47, NL-1105BA Amsterdam, The Netherlands

3: Department of Psychology, University of Amsterdam, Nieuwe Achtergracht 116, NL-1018 WV, Amsterdam, The Netherlands

**Supplementary Information**

**Supplementary Methods**

**Further information on the experimental procedure.**

For the Agent-LOOP (experiments 1 and 3) both participants arrived at the same time, were led to the same room and instructed about the task by the experimenter. After signing the informed consent forms, they went to separate rooms to fill out demographic and self-esteem questionnaires and to practice the estimation task. Each room was equipped with a desktop computer and participants were told that the two computers were connected. While the other participant completed the estimation question, participants could see the estimation question, but not the answer given by the other participant. For the private version of the Agent-LOOP the EXP rating was private as well and could not be seen by the other participant.

For all three experiments participants were asked to fill in personality questionnaires and a post-experimental questionnaire. Before leaving, all participants received compensation for their participation, including an additional 6 cents per trial promised for “accurate” EXP ratings, and were debriefed about the study. The total duration of the experiments, including post-experiment questionnaires, was 1.5 to 2 hours.

The sample recruited for experiment 2 was part of another project assessing the impact of stress on self-related learning. All participants therefore completed a simple reading task prior to the estimation task, which served as a control condition for a stress task. This took approximately 45 minutes and during the whole task period cortisol samples were collected.

**Detailed information on the model space.**

To see whether a learning model can capture the participants’ behavior and allows us to summarize the data using principled parameters such as learning rates, we performed a model comparison (see **Figure 3**). Our model space contained three main models varying with regards to their assumptions about biased updating behavior when learning about the self (see **Figure 3**). The simplest learning model used one single learning rate for the whole behavioral time course for each participant, thus not assuming any learning biases [EXP_t+1_=EXP_t_ + α_Uni_ PE_t_ , while PE_t=_FB_t_ - EXP_t_; Unity Model]. The second model, the Ability Model, contained a separate learning rate for each of the ability conditions, assuming that participants would show different updating behavior in the High Ability condition (α_HA_) vs Low Ability condition (α_LA_). The third model, the Valence Model, included separate learning rates for positive PEs (α_PE+_) vs negative PEs (α_PE-_) across both ability conditions, thus suggesting that the valence (positive vs negative) of the PE biases self-related learning rather than the ability condition itself. In the Agent-LOOP task (experiments 1 and 3) the distinction between learning about oneself vs another person was introduced as a second factor in the model space resulting in three additional models. Model 4 corresponded to the Unity Model with separate learning rates for the self (α_Uni(S)_) and the other person (α_Uni(O)_). Model 5 was the extension of the Ability Model distinguishing between learning about the self (α_HA(S)_, α_LA(S)_) and the other person (α_HA(O)_, α_LA(O)_), resulting in four different learning rates. Model 6 extended the Valence Model by separate learning rates for oneself (α_PE+(S)_, α_PE-(S)_) and the other person (α_PE+(O)_, α_PE-(O)_).

All models in the complete model space not only differed with regard to the learning rates as described in the main manuscript but also with regard to the initial belief about the own and the other participant’s performance. In addition to the learning rates for all 3/ 6 learning models described in the main manuscript we either fitted parameters for the initial belief about the own and the other participant’s performance, separately (Models 1-3 for the Audience-LOOP and Models 1-6 for the Agent-LOOP) or combined for both ability conditions (Models 5-7 for the Audience-LOOP and Models 8-13 for the Agent-LOOP), or used the initial performance expectation ratings as fixed starting values (Models 8-10 for the Audience-LOOP and Models 14-19 for the Agent-LOOP), resulting in 18 learning models for experiments 1 and 3, and 9 learning models for experiment 2.

To test if the participants’ EXP ratings could be better explained in terms of prediction error learning as compared to stable assumptions in each ability condition, we included a simple Mean Model with a mean value for each task condition. The Mean Models were numbered Model 4 for the Audience-LOOP und Model 7 for the Agent-LOOP to keep the numbering in the main manuscript consitent. For the Audience-LOOP one mean value for the High Ability condition was estimated and one for the Low Ability condition. For the Agent-LOOP four mean values were estimated for the Agent (Self vs Other) x Ability condition (High vs Low). PSIS-LOO scores for all models are reported in **Supplementary Tables S2 and S3.**

**Supplementary Results**

**Additional model free behavioral analyses.**

The combined analysis of the public and private Agent-LOOP (experiment 1 and 3) confirmed the results of the Audience-LOOP by showing that Audience did not have any significant effects also with regards to the additional Agent condition (main effect of Audience: *F_(1,50)_*=0.61, *p*=.439; Audience x Ability condition: *F_(1,50)_*=0.53, *p*=.472; Audience x Agent condition: *F_(1,50)_*=0.31, *p*=.581; Audience x Ability condition x Agent condition: *F_(1,50)_*=0.61, *p*=.440; Audience x Ability condition x Trial: *F_(24,1200)_*=0.47, *p*=.987; Audience x Agent condition x Trial: *F_(24,1200)_*=0.76, *p*=.794; Audience x Ability condition x Agent condition x Trial: *F_(24,1200)_*=0.65, *p*=.903). The remaining effects stayed consistent with the separate analyses of experiment 1 (main effect Ability condition: *F_(1,50)_*=386.16, *p*<.001; Trial x Ability condition: *F_(24,1200)_*=66.55, *p*<.001; main effect of Agent condition: *F_(1,50)_*=32.09, *p*<.001; Agent condition x Ability condition: *F_(1,50)_*=6.97, *p*=.011; Trial x Agent condition x Ability condition interaction: *F_(24,1200)_*=1.43, *p*=.083).

**Model free behavioral analyses on the extended sample.**

We replicated the model free behavioral analysis on the EXP ratings including the four participants that had been excluded after model fitting and the results matched the results of the smaller sample. For the Agent-LOOP in experiment 1 the Trial x Ability condition x Agent condition ANOVA again revealed a significant main effect of Ability condition (*F_(1,23)_*=225.68, *p*<.001) and Agent condition (*F_(1,23)_*=17.60, *p*<.001) and interactions of Trial x Ability condition (*F_(24,552)_*=31.12, *p*<.001) and Agent condition x Ability condition (*F_(1,23)_*=5.42, *p*=.029). For the Audience-LOOP (experiment 2) we also found a significant main effect of Ability condition (*F_(1,59)_*=262.9, *p*<.001) and interaction of Trial x Ability condition (*F_(29,1711)_*=42.66, *p*<.001), while there was no significant impact of the Audience on EXP ratings (main effect of Audience: *F_(1,59)_*=0.20, *p*=.655; Audience x Ability condition: *F_(1,59)_*=0.32, *p*=.571; Audience x Ability condition x Trial: *F_(29,1711)_*=1.08, *p*=.357). For the public version of the Agent-LOOP in experiment 3 the results including all participants also stayed the same (main effect of Ability condition: *F_(1,29)_*=194.68, *p*<.001; Agent condition: *F_(1,29)_*=20.24, *p*<.001; interaction of Trial x Ability condition: *F_(24,672)_*=39.92, *p*<.001; no significant interaction of Agent condition x Ability condition: *F_(1,29)_*=2.19, *p*=.149). The results of the combined analysis of the public and private Agent-LOOP (experiment 1 and 3) stayed the same as well (main effect Ability condition: *F_(1,52)_*=406.92, *p*<.001; Trial x Ability condition: *F_(24,1248)_*=69.02, *p*<.001; main effect of Agent condition: *F_(1,52)_*=35.52, *p*<.001; Agent condition x Ability condition: *F_(1,52)_*=7.72, *p*=.008; Trial x Agent condition x Ability condition interaction: *F_(24,1248)_*=1.34, *p*=.126; Audience related effects: main effect of Audience: *F_(1,52)_*=0.61, *p*=.439; Audience x Ability condition: *F_(1,52)_*=0.27, *p*=.607; Audience x Agent condition: *F_(1,52)_*=0.49, *p*=.488; Audience x Ability condition x Agent condition: *F_(1,52)_*=0.82, *p*=.370; Audience x Ability condition x Trial: *F_(24,1248)_*=0.52, *p*=.975; Audience x Agent condition x Trial: *F_(24,1248)_*=0.72, *p*=.836; Audience x Ability condition x Agent condition x Trial: *F_(24,1248)_*=0.73, *p*=.825).

**Posterior predictive checks: Behavioral analyses on the predicted data.**

To assess whether our winning model captured the core effects in our model free analysis, we let the parametrized winning model predict the time course of EXP for each participant, and compared these model predictions against the actual data (see **Figure 2**). **Figure 2** visually confirms the ability of the model to capture the observed data despite its small number of parameters. We repeated the behavioral analyses we had done on the actual behavioral data on the predicted data. For the Agent-LOOP in experiment 1 the Trial x Ability condition x Agent condition ANOVA on the predicted data revealed a significant main effect of Ability condition (*F_(1,22)_*=273.80, *p*<.001) and an interaction of Trial x Ability condition (*F_(24,528)_*=199.29, *p*<.001). Again, there was no significant interaction of Trials x Agent condition x Ability condition (*F_(24,528)_*=0.72, *p*=.838) but the main effect of Agent condition (*F_(1,22)_*=17.09, *p*<.001) and the Agent condition x Ability condition interaction (*F_(1,22)_*=5.40, *p*=.030) confirmed the negative bias for self- vs other-related evaluations we found in the behavioral data. For the Audience-LOOP (experiment 2) we also found a significant main effect of Ability condition (*F_(1,57)_*=305.45, *p*<.001) and interaction of Trial x Ability condition (*F_(29,1653)_*=210.43, *p*<.001) indicating that the participants’ belief updating was reflected in the predicted data, while there was no significant impact of the Audience on EXP ratings (main effect of Audience: *F_(1,57)_*=0.07, *p*=.789; Audience x Ability condition: *F_(1,57)_*=0.62, *p*=.436; Audience x Ability condition x Trial: *F_(29,1653)_*=1.23, *p*=.187). For the public version of the Agent-LOOP in experiment 3 the results of the predicted data were also comparable to the actual behavioral data (main effect of Ability condition: *F_(1,28)_*=222.63, *p*<.001; interaction of Trial x Ability condition: *F_(24,672)_*=227.63, p<.001; main effect of Agent condition: *F_(1,28)_*=20.02, *p*<.001; no significant interaction of Agent condition x Ability condition: *F_(1,28)_*=1.19, *p*=.285; and no Trial x Agent condition x Ability condition: *F_(24,528)_*=0.06, *p*>.999). Repeating the behavioral analysis we had done on the model free data onto the predictions thus confirmed that it recapitulates the tendency towards more negative performance expectations for the other that was core to our data.

**Additional results on learning rates and parameter correlations.**

### Across all experiments learning rates were significantly greater than zero (all ps<.001) indicating that, as intended, participants updated their self-related expectations according to the provided feedback (see also **Figure 4**). This is supported by model comparisons favoring the learning models over the Mean Model that does not assume prediction error learning during the experiments (see **Supplementary Tables S2 and S3**).

We calculated Pearson correlations between the parameters within the winning model and for each of the experiments and found no significant correlations between learning rates and starting values (*p*>.05, with p-values corrected for multiple comparisons: *p*=.05/28=.002 (Agent-LOOP; experiment 1/ 3) and *p*=.05/6=.008 (Audience-LOOP). This suggests that learning rates were neither strongly biased nor restricted by participants’ estimated EXP starting level and starting levels alone are unlikely to explain interindividual differences in learning behavior. Learning rates were positively correlated within the winning model, specifically learning rates within the Self and the Other condition (see **Supplementary** **Tables S4-S6**), which makes it unlikely that differences in learning rates between positive and negative PEs would be induced by anti-correlations induced by the model fitting procedure.

**Supplementary Tables**

| *Table S1. Sample characteristics* | | | | | | | | | |  |  |  |
| --- | --- | --- | --- | --- | --- | --- | --- | --- | --- | --- | --- | --- |
|  | | **Experiment 1** | |  | **Experiment 2** | | | | |  | **Experiment 3** | |
|  |  |  |  |  | Private | |  | Public | |  |  |  |
|  |  | Mean | SD |  | Mean | SD |  | Mean | SD |  | Mean | SD |
|  |  |  |  |  |  |  |  |  |  |  |  |  |
| **Age** |  | 23.75 | 3.22 |  | 22.24 | 3.05 |  | 22.58 | 2.69 |  | 21.70 | 3.33 |
| **Self-esteem** | | 6.09 | 0.89 |  |  |  |  |  |  |  | 6.01 | 0.89 |
| **SIAS** | | 2.04 | 0.47 |  | 1.97 | 0.55 |  | 2.01 | 0.49 |  | 2.01 | 0.52 |
|  |  |  |  |  |  |  |  |  |  |  |  |  |
| *Note.* Sample characteristics for the three experiments. SD = standard deviation; SIAS = averaged score on the Social Interaction Anxiety Scale; Experiment 1: N=24; Experiment 2: N(Private)=30; N(Public)=31; Experiment 3: N=30. | | | | | | | | | | | | |
|  |  |  |  |  |  |  |  |  |  |  |  |  |
|  |  |  |  |  |  |  |  |  |  |  |  |  |
|  |  |  |  |  |  |  |  |  |  |  |  |  |
|  |  |  |  |  |  |  |  |  |  |  |  |  |

| *Table S2. Model comparisons for the Agent-LOOP task* | | | | | | | | | | | | | | | | | |
| --- | --- | --- | --- | --- | --- | --- | --- | --- | --- | --- | --- | --- | --- | --- | --- | --- | --- |
| Model | | | | | | LOO | LOO-SE | | LOO-Diff (SE-Diff) | | | | % of k̂ > 0.7 | | No. Est. Parameters |  |  |
|  |  |  |  |  |  |  |  |  |  |  |  |  |  |  |  |  |  |
|  | |  | |  | |  |  | |  | | |  | | |  | | |
| ***Learning Models*** | | | | | |  |  | |  | | | |  | |  |  |  |
| **Estimated IV for Self vs Other x High vs Low Ability** | | | | | | | | | | | |  | | |  | | |
|  | | **Self = Other** | | | |  |  | |  | | | |  | |  |  |  |
|  | |  | | Unity Model (M1) | | -2380.1 | 247.8 | | 135.4 (63.7) | | | 0.1 | | | 5 | | |
|  | |  | | Ability Model (M2) | | -2336.5 | 261.5 | | 91.7 (42.4) | | | 0.3 | | | 6 | | |
|  | |  | | Valence Model (M3) | | -2320.5 | 259.0 | | 75.7 (49.4) | | | 0.2 | | | 6 | | |
|  | | **Self ≠ Other** | | | |  |  | |  | | | |  | |  |  |  |
|  | |  | | Unity Model (M4) | | -2376.2 | 254.8 | | 131.5 (54.6) | | | 0.4 | | | 6 | | |
|  | |  | | Ability Model (M5) | | -2330.7 | 263.3 | | 85.9 (42.8) | | | 1.2 | | | 8 | | |
|  | |  | | Valence Model (M6) | | -2244.8 | 283.5 | | - | | | 0.3 | | | 8 | | |
| **Estimated IV for Self vs Other across ability conditions** | | | | | | | | | | | |  | | |  | | |
|  | | **Self = Other** | | | |  |  | |  | | | |  | |  |  |  |
|  | |  | | Unity Model (M8) | | -2516.4 | 237.8 | | 271.6 (88.5) | | | 0.0 | | | 3 | | |
|  | |  | | Ability Model (M9) | | -2409.8 | 241.9 | | 165.0 (69.6) | | | 0.1 | | | 4 | | |
|  | |  | | Valence Model (M10) | | -2428.1 | 244.6 | | 183.3 (73.0) | | | 0.0 | | | 4 | | |
|  | | **Self ≠ Other** | | | |  |  | |  | | | |  | |  |  |  |
|  | |  | | Unity Model (M11) | | -2440.9 | 237.0 | | 196.1 (87.5) | | | 0.1 | | | 4 | | |
|  | |  | | Ability Model (M12) | | -2363.5 | 250.3 | | 118.8 (63.3) | | | 0.8 | | | 6 | | |
|  | |  | | Valence Model (M13) | | -2232.7 | 261.7 | | -12.1 (32.2) | | | 0.1 | | | 6 | | |
| **Fixed IV** | | | | | |  |  | |  | | | |  | |  |  |  |
|  | | **Self = Other** | | | |  |  | |  | | | |  | |  |  |  |
|  | |  | | Unity Model (M14) | | -2943.3 | 244.4 | | 698.5 (126.4) | | | 0.0 | | | 1 | | |
|  | |  | | Ability Model (M15) | | -2763.9 | 240.5 | | 519.1 (106.9) | | | 0.1 | | | 2 | | |
|  | |  | | Valence Model (M16) | | -2765.2 | 247.9 | | 520.5 (101.5) | | | 0.0 | | | 2 | | |
|  | | **Self ≠ Other** | | | |  |  | |  | | | |  | |  |  |  |
|  | |  | | Unity Model (M17) | | -2840.0 | 249.4 | | 595.3 (109.8) | | | 0.1 | | | 2 | | |
|  | |  | | Ability Model (M18) | | -2566.4 | 251.5 | | 321.7 (78.3) | | | 0.6 | | | 4 | | |
|  | |  | | Valence Model (M19) | | -2453.8 | 270.3 | | 209.0 (51.7) | | | 0.1 | | | 4 | | |
|  | |  | |  | |  |  | |  | | |  | | |  | | |
| ***No Learning*** | | | | | |  |  | |  | | | |  | |  |  |  |
|  | |  | | Mean Model (M7) | | -2953.6 | 190.3 | | 708.9 (123.3) | | | 0.0 | | | 4 | | |
|  | |  | |  | |  |  | |  | | |  | | |  | | |
| *Note.* LOO = sum PSIS-LOO, approximate leave-one-out cross-validation (LOO) using Pareto-smoothed importance sampling (PSIS); LOO-SE = Standard error of PSIS-LOO; LOO-Diff (SE-Diff) = Difference in expected predictive accuracy (PSIS-LOO) for all models from the model with the highest PSIS-LOO (Valence Model) and standard errors of differences; percentage of*k̂ -* estimated shape parameters of the generalized Pareto distribution - exceeding 0.7 (all according to Vehtari et al. 2017); No. Est. Parameters = number of estimated parameters in the model. IV = initial parameter values for the performance expectations. | | | | | | | | | | | | | | | | | |
|  |  |  |  |  |  |  |  |  |  |  |  |  |  |  |  |  |  |
|  |  |  |  |  |  |  |  |  |  |  |  |  |  |  |  |  |  |
|  |  |  |  |  |  |  |  |  |  |  |  |  |  |  |  |  |  |
|  |  |  |  |  |  |  |  |  |  |  |  |  |  |  |  |  |  |
| *Table S3. Model comparisons for the Audience-LOOP task* | | | | | | | | | | | | | | | | |  |
| Model | | | | | PSIS-LOO | | | LOO-SE | | LOO-Diff (SE-Diff) | % of k̂>0.7 | | | No. Est. Parameters | | |  |
|  |  |  |  |  |  |  |  |  |  |  |  |  |  |  |  |  |  |
|  |  | |  | |  | | |  | |  |  | | |  | | |  |
| ***Learning Models*** | | | | |  | | |  | |  |  | | |  | | |  |
| **Estimated IV for High vs Low Ability** | | | | | | | | | | |  | | |  | | |  |
|  |  | | Unity Model (M1) | | -708.2 | | | 145.1 | | 213.1 (35.8) | 0.1 | | | 3 | | |  |
|  |  | | Ability Model (M2) | | -570.2 | | | 150.0 | | 75.0 (26.8) | 0.3 | | | 4 | | |  |
|  |  | | Valence Model (M3) | | -495.2 | | | 150.9 | | - | 0.1 | | | 4 | | |  |
| **Estimated IV across ability conditions** | | | | | | | | | | |  | | |  | | |  |
|  |  | | Unity Model (M5) | | -943.0 | | | 134.6 | | 447.9 (79.9) | 0.0 | | | 2 | | |  |
|  |  | | Ability Model (M6) | | -733.6 | | | 141.4 | | 238.5 (57.7) | 0.2 | | | 3 | | |  |
|  |  | | Valence Model (M7) | | -623.6 | | | 142.9 | | 128.5 (61.2) | 0.1 | | | 3 | | |  |
| **Fixed IV** | | | | | | | | | |  |  | | |  | | |  |
|  |  | | Unity Model (M8) | | -1387.9 | | | 177.5 | | 892.8 (149.6) | 0.0 | | | 1 | | |  |
|  |  | | Ability Model (M9) | | -1177.8 | | | 206.4 | | 682.7 (170.9) | 0.7 | | | 2 | | |  |
|  |  | | Valence Model (M10) | | -975.8 | | | 163.8 | | 480.6 (107.4) | 0.0 | | | 2 | | |  |
|  |  | |  | |  | | |  | |  |  | | |  | | |  |
| ***No Learning*** | | | | |  | | |  | |  |  | | |  | | |  |
|  |  | | Mean Model (M4) | | -1189.5 | | | 124.9 | | 694.4 (61.3) | 0.0 | | | 2 | | |  |
|  |  | |  | |  | | |  | |  |  | | |  | | |  |
| *Note.* LOO = sum PSIS-LOO, approximate leave-one-out cross-validation (LOO) using Pareto-smoothed importance sampling (PSIS); LOO-SE = Standard error of PSIS-LOO; LOO-Diff (SE-Diff) = Difference in expected predictive accuracy (PSIS-LOO) for all models from the model with the highest PSIS-LOO (Valence Model) and standard errors of differences; percentage of*k̂ -* estimated shape parameters of the generalized Pareto distribution - exceeding 0.7 (all according to Vehtari et al. 2017); No. Est. Parameters = number of estimated parameters in the model. IV = initial parameter values for the performance expectations. | | | | | | | | | | | | | | | | |  |
|  |  |  |  |  |  |  |  |  |  |  |  |  |  |  |  |  |  |
|  |  |  |  |  |  |  |  |  |  |  |  |  |  |  |  |  |  |
|  |  |  |  |  |  |  |  |  |  |  |  |  |  |  |  |  |  |
|  |  |  |  |  |  |  |  |  |  |  |  |  |  |  |  |  |  |
|  |  |  |  |  |  |  |  |  |  |  |  |  |  |  |  |  |  |
|  |  |  |  |  |  |  |  |  |  |  |  |  |  |  |  |  |  |
|  |  |  |  |  |  |  |  |  |  |  |  |  |  |  |  |  |  |

*Table S4. Parameter Correlations for Experiment 1*

|  |  | Model Parameter | | | | | | | |
| --- | --- | --- | --- | --- | --- | --- | --- | --- | --- |
|  |  | IV_S+_ | IV_S-_ | IV_O+_ | IV_O-_ | α_PE+(S)_ | α_PE-(S)_ | α_PE+(O)_ | α_PE-(O)_ |
| Model Parameter | IV_S+_ |  |  | .31 | .12 | .26 | .06 | .12 | .08 |
|  | IV_S-_ | .40 |  | .11 | .31 | .20 | .20 | .15 | .17 |
|  | IV_O+_ | .31 | .11 |  | -.02 | .11 | .06 | .16 | .33 |
|  | IV_O-_ | .12 | .31 | -.02 |  | .00 | -.02 | -.15 | -.11 |
|  | α_PE+(S)_ | .26 | .20 | .11 | .00 |  | .67* | .71* | .74* |
|  | α_PE-(S)_ | .06 | .20 | .06 | -.02 | .67* |  | .62* | .82* |
|  | α_PE+(O)_ | .12 | .15 | .16 | -.15 | .71* | .62* |  | .84* |
|  | α_PE-(O)_ | .08 | .17 | .33 | -.11 | .74* | .82* | .84* |  |

*Table S5. Parameter Correlations for Experiment 2*

|  | Model Parameter | | | | | | | | | | |
| --- | --- | --- | --- | --- | --- | --- | --- | --- | --- | --- | --- |
|  | Private | | | | |  | Public | | | | |
|  |  |  |  |  |  |  |  |  |  |  |  |
| Model Parameter |  | IV_+_ | IV_-_ | α_PE+(S)_ | α_PE-(S)_ |  |  | IV_+_ | IV_-_ | α_PE+(S)_ | α_PE-(S)_ |
|  | IV_+_ |  | .64* | .35 | .14 |  | IV_+_ |  | .30 | .02 | -.14 |
|  | IV_-_ | .64* |  | .30 | .21 |  | IV_-_ | .30 |  | .07 | -.05 |
|  | α_PE+_ | .35 | .30 |  | .66* |  | α_PE+_ | .02 | .07 |  | .72* |
|  | α_PE-_ | .14 | .21 | .66* |  |  | α_PE-_ | -.14 | -.05 | .72* |  |
|  |  |  |  |  |  |  |  |  |  |  |  |

*Table S6. Parameter Correlations for Experiment 3*

|  |  | Model Parameter | | | | | | | |
| --- | --- | --- | --- | --- | --- | --- | --- | --- | --- |
|  |  | IV_S+_ | IV_S-_ | IV_O+_ | IV_O-_ | α_PE+(S)_ | α_PE-(S)_ | α_PE+(O)_ | α_PE-(O)_ |
| Model Parameter | IV_S+_ |  | .04 | .21 | -.15 | -.27 | -.36 | .14 | -.02 |
|  | IV_S-_ | .04 |  | -.37 | -.15 | .00 | -.21 | -.21 | -.21 |
|  | IV_O+_ | .21 | -.37 |  | -.05 | -.13 | .19 | -.16 | -.19 |
|  | IV_O-_ | -.15 | -.15 | -.05 |  | .03 | -.07 | -.07 | .16 |
|  | α_PE+(S)_ | -.27 | .00 | -.13 | .03 |  | .65* | .49 | .46 |
|  | α_PE-(S)_ | -.36 | -.21 | .19 | -.07 | .65* |  | .30 | .31 |
|  | α_PE+(O)_ | .14 | -.21 | -.16 | -.07 | .49 | .30 |  | .84* |
|  | α_PE-(O)_ | -.02 | -.21 | -.19 | .16 | .46 | .31 | .84* |  |

*Note.* Parameter correlations for the three experiments. The correlation tables include the initial parameter values for the performance expectations (IV) in the high ability condition (IV_+_) and for the low ability condition (IV_-_) and learning rates (α) for positive prediction errors (α_PE+_) and for negative prediction errors (α_PE-_). For experiments 1 and 3, as depicted in Table S4 and S6, parameters are separated for the Self condition (IV_S+_, IV_S-_, α_PE+(S)_, α_PE-(S)_) and the Other condition (IV_O+_, IV_O-_, α_PE+(O)_, α_PE-(O)_). * indicates correlations with p-values < .05, corrected for multiple comparison as described above.
